# Surface-Driven Electron Localization and Defect Heterogeneity in Ceria

**DOI:** 10.1021/jacs.5c10679

**Published:** 2025-09-09

**Authors:** Xingfan Zhang, Akira Yoko, Yi Zhou, Woongkyu Jee, Alvaro Mayoral, Taifeng Liu, Jingcheng Guan, You Lu, Thomas W. Keal, John Buckeridge, Kakeru Ninomiya, Maiko Nishibori, Susumu Yamamoto, Iwao Matsuda, Tadafumi Adschiri, Osamu Terasaki, Scott M. Woodley, C. Richard A. Catlow, Alexey A. Sokol

**Affiliations:** † Kathleen Lonsdale Materials Chemistry, Department of Chemistry, 4919 University College London, London WC1H 0AJ, U.K.; ‡ WPI-Advanced Institute for Materials Research (WPI-AIMR), 13101 Tohoku University, 2-1-1 Katahira, Aoba-ku, Sendai 980-8577, Japan; § International Center for Synchrotron Radiation Innovation Smart (SRIS), 13101 Tohoku University, 468-1, Aramaki-Aza-Aoba, Aoba-ku, Sendai 980-8572, Japan; ∥ Centre for High-Resolution Electron Microscopy (CℏEM), School of Physical Science and Technology and Shanghai Key Laboratory of High-Resolution Electron Microscopy, 387433 ShanghaiTech University, Shanghai 201210, P. R. China; ⊥ Scientific Computing Department, STFC Daresbury Laboratory, Warrington, Cheshire WA4 4AD, U.K.; # School of Engineering and Design, 4914 London South Bank University, London SE1 OAA, U.K.; ∇ Instituto de Nanociencia y Materiales de Aragón (INMA), CSIC-Universidad de Zaragoza, Zaragoza 50009, Spain; ○ National & Local Joint Engineering Research Center for Applied Technology of Hybrid Nanomaterials, Henan University, Kaifeng 475004, China; ◆ New Industry Creation Hatchery Center, 13101 Tohoku University, Sendai 980-8579, Japan; ¶ Institute of Multidisciplinary Research for Advanced Materials, 13101 Tohoku University, Sendai 980-8577, Japan; & The Institute for Solid State Physics, The University of Tokyo, Kashiwa, Chiba 277-8581, Japan; ● School of Chemistry, Cardiff University, Park Place, Cardiff CF10 1AT, U.K.

## Abstract

The exceptional performance of ceria (CeO_2_) in catalysis and energy conversion is fundamentally governed by its defect chemistry, particularly oxygen vacancies. The formation of each oxygen vacancy (V_O_^••^) is assumed to be compensated by two localized electrons on cations (Ce^3+^). Here, we show by combining theory with experiment that while this 1 V_O_^••^: 2Ce^3+^ ratio accounts for the global charge compensation, it does not apply at the local scale, particularly in nanoparticles. Hybrid quantum mechanical/molecular mechanical (QM/MM) defect calculations, together with synchrotron X-ray photoelectron spectroscopy (XPS) measurements, show that electrons have a strong preference to localize and segregate on surfaces, which can overcome the trapping force from the V_O_^••^ sites in the bulk. At a given Fermi level, the surface V_O_^••^ tends to trap more electrons than those in bulk, resulting in a higher Ce^3+^ to V_O_^••^ ratio on surfaces than that in the bulk, driven by the preferential localization of electrons and enhanced V_O_^••^–Ce^3+^coupling. Large-scale unbiased Monte Carlo simulations on ceria nanoparticles confirmed this trend and further show that the surface segregation of electrons is more pronounced at low reduction levels and in smaller nanoparticles. In highly reduced ceria nanoparticles, however, the enhanced repulsive interactions lead to a less significant extent of defect heterogeneity or even reverse the location preference of defects in some nanoparticles. Our findings underscore the need to consider both the overall nonstoichiometry and local defect behavior in easily reducible oxides, with direct relevance to their performance in catalytic and energy applications.

## Graphical abstract

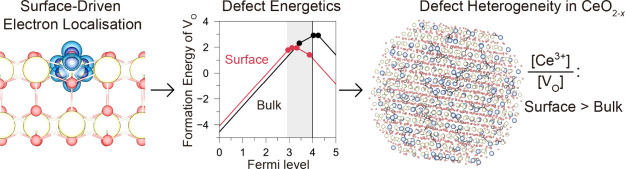

## 1 Introduction

Oxygen vacancies have long been recognized as key components in ceria (CeO_2_) for enhancing its technological applications across different fields.^1^ In bulk, oxygen vacancies promote ionic conduction in ceria, which serves as the electrolyte in solid-oxide fuel cell (SOFC) applications.^2^ On surfaces, oxygen vacancies become the active sites for a broad range of chemical reactions, as well as interacting with supported metal clusters and single-atom catalysts to improve their catalytic activity.^3^ Therefore, understanding and controlling the defect chemistry in ceria is the key to optimizing its performance in various applications.

Upon formation of an oxygen vacancy (V_O_^••^) in ceria, to preserve charge neutrality, the two excess electrons localize on the Ce sites and form small polarons (Ce^3+^, or Ce_Ce_′ in Kröger–Vink notation^4^), i.e., $O_{O}^{\times }\to V_{O}^{••}+\frac{1}{2}O_{2}\left(g\right)+2{\mathrm{Ce}}_{\mathrm{Ce}}^{\prime }$ . This defect process results in deviation from the ideal stoichiometry (CeO_2–*x*_). Although the nonstoichiometric nature of ceria is well established, the spatial correlation of V_O_^••^ and Ce_Ce_′ polarons in ceria remains less clear. First, the excess electrons in ceria were found not to favor the localization at the nearest neighbor (NN) sites of V_O_^••^, but rather, at least one electron localizes at the next-nearest neighbor (NNN) site. Such a preference has been demonstrated both in bulk^5–7^ and on low-index surfaces^8,9^ by different levels of theoretical calculations, and observed on the (111) surface using scanning tunnelling microscope (STM).^10^ However, given the close stability of different polaron configurations^5–7^ and the low hopping barrier of Ce_Ce_′ polarons,^11^ the actual polaron distribution is likely to be dynamically complex.

The defect chemistry in ceria differs significantly between the bulk and surface regions. In polycrystalline ceria, enhanced electronic conductivity and reduced ionic conductivity were observed with a decreased grain size. To account for this behavior, a space-charge model was proposed by Tschöpe^12^ and Kim and Maier,^13^ which predicts a positive electrostatic potential near the surface region. This space-charge region favors the accumulation of electrons and depletion of V_O_^••^ near surfaces. However, thermogravimetric analysis, temperature-programmed reduction, and oxygen titration measurements have shown enhanced reduction and nonstoichiometry in surface regions of ceria.^14,15^ Density functional theory (DFT) calculations also showed that the formation energies of charge-neutral oxygen vacancies ([V_O_^••^ + 2Ce_Ce_′]^×^) near the surfaces are significantly lower than those in the bulk.^9,16^ To explain the enhanced surface oxygen deficiency, Tschöpe^17^ further treated surfaces and grain boundaries as thermodynamically distinct phases, proposing that oxygen vacancy segregation itself generates the space charge, rather than being its consequence. Sheldon and Shenoy^18^ considered the stress induced by Ce^3+^ in standard space-charge models, which significantly affects thermodynamic defect equilibrium. These studies show that a comprehensive understanding of defect chemistry in ceria requires explicit consideration of the defect formation mechanism, its interactions, and the resulting spatial distributions under varying thermodynamic and structural constraints.

Nanosized ceria shows an enhanced extent of nonstoichiometry, primarily attributed to surface effects.^14,15^ Various ceria nanostructures, including polyhedra, nanorods, nanocubes, nanospheres, and nanosheets, can be controllably synthesized,^19^ whose morphological differences should have a large impact on the spatial distribution of defects and their reactivity. Neutron scattering measurements on ceria nanorods provided direct evidence for heterogeneous defect distributions in nanorods, where Frenkel-type oxygen defects ([V_O_^••^ + O_i_″]^×^) are predominant in the bulk, while a partially reduced Ce_3_O_5+*x*_ pattern appears on the surface.^20^ Lawrence et al.^21^ compared the defect structures and CO oxidation activity across bulk ceria, nanorods, and nanoparticles. Upon activation at 400 °C under 0.1 Torr, nanorods and nanoparticles showed a significant increase in the concentration of Ce^3+^, while bulk samples remained unchanged. The low-pressure treated nanorods outperform other samples in CO oxidation, underscoring the critical role of morphology and size in governing defect behavior and catalytic performance.

Elucidating defect structures in easily reducible oxides remains a significant challenge in both computational and experimental studies due to the vast configurational space of defects.^22^ Experimentally, oxygen vacancies within oxide materials can be directly imaged with atom probe tomography,^23^ scanning transmission electron microscopy (STEM),^24^ atomic force microscopy (AFM),^25^ and STM.^26^ However, quantifying vacancy distribution within the sample and relating it to physical or chemical properties remains a challenging task.^27^ Computationally, an accurate description of the defects and electronic properties of cerium oxides is difficult. While the Hubbard *U* correction (*U*_Ce 4f_ *=* 4.5–6 eV*)* can partially address the localization problem of Ce 4f electrons arising from local and semilocal exchange-correlation functionals in DFT calculations, accurate modeling of cerium oxides requires nonlocal hybrid functionals.^16^ DFT calculations using hybrid functionals remain computationally demanding, especially in plane-wave codes. Although localized basis sets reduce the cost,^28^ modeling complex interacting defects in bulk or nanoparticle systems remains challenging due to the unfavorable scaling of the computational cost of such calculations with the number of atoms in the simulation cell *N*, formally ∼*N* ^4^, and the need to employ large supercells for charged defects to minimise errors arising from artificial image–image interactions. These systems are more effectively addressed by using reliable interatomic potentials that support large-scale simulations with retained physical accuracy.

To overcome these challenges, we have recently developed a robust shell-model interatomic potential for accurate modeling of charged defects in ceria, which substantially reduces the computational cost when extending to large-scale systems, while maintaining high-level accuracy comparable to hybrid DFT calculations and experimental measurements.^7^ Moreover, this interatomic potential can be integrated into hybrid quantum mechanical/molecular mechanical (QM/MM) embedded-cluster models, which support accurate and efficient defect energy calculations at the dilute limit with hybrid DFT functionals, avoiding the problem of spurious image–image interactions among charged species in conventional periodic supercell models.^29,30^ These techniques have been successfully employed to understand the variable band edge positions and work function in ceria originating from surface and defect structures.^31,32^

In this work, we integrate several advanced theoretical modeling and experimental approaches to investigate defect formation, interactions, and spatial distributions in ceria. Our findings reveal a new characteristic of ceria: defect heterogeneity driven by preferential surface electron localization. Our findings have implications for other reducible oxides.

## 2 Results

### 2.1 Intrinsic Defect Chemistry in Bulk CeO_2_

In our previous work,^7^ we employed the hybrid QM/MM embedded-cluster approach (Figure 1a) to calculate the formation of defects in bulk ceria, with the BB1K^33^ hybrid meta-GGA DFT functional to describe electronic localization accurately in the QM region. Here, based on the calculated defect formation energies (Figure S1a,b), we further calculated the equilibrium-state concentrations of defects and charge carriers along with the self-consistent Fermi level in CeO_2_ under different thermal conditions (from 100 to 1600 K) and oxygen partial pressures (1 and 10^–8^ atm), using the SC-FERMI^34^ code (Figure S1c,d). CeO_2_ shows an intrinsic n-type characteristic with V_O_ (Figure S1c) as the dominant defect type, where their concentration exceeds that of other types of point defects by several orders of magnitude.^7^ The equilibrium-state stoichiometry of CeO_2–*x*_, derived from the calculated defect concentrations (Figure S2) at the dilute limit, aligns well with experimental data under mildly reduced conditions (*x* < 0.05) and temperatures below 1200 K. At higher reduction levels, notable deviations emerge, and the prediction based solely on noninteracting defect energies significantly underestimates the extent of nonstoichiometry. Indeed, as reported by Bishop et al.,^35^ measurements on ceria samples with varying surface areas can yield substantially different nonstoichiometry values under identical environmental conditions. Moreover, Monte Carlo simulations and free energy calculations by Grieshammer et al.^36,37^ also showed that defect–defect interactions reduce the average formation energy of multiple vacancies. These results highlight the importance of explicitly accounting for defect interactions and surface effects.

**Figure 1:**
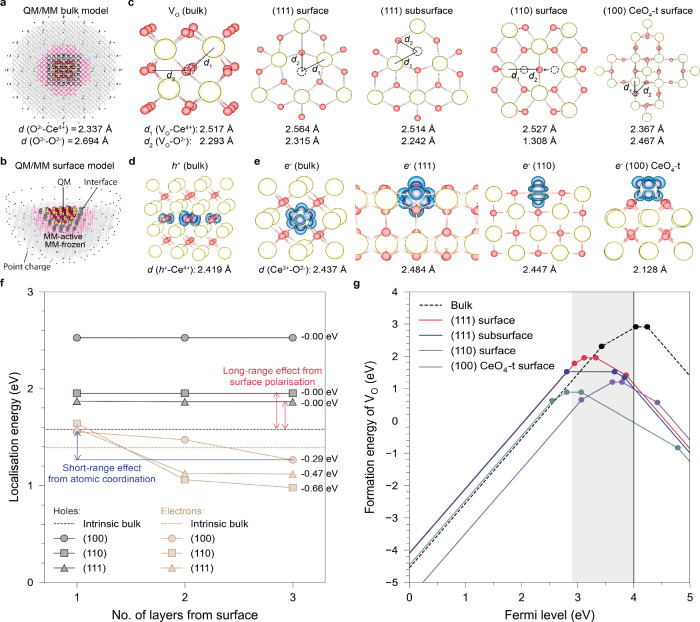
Preferential localization of electrons on surfaces of CeO_2_ calculated with hybrid QM/MM embedded-cluster models. Hybrid QM/MM embedded-cluster model for studying localized states in (a) bulk and (b) surface systems. (c) Optimized configurations of oxygen vacancies formed in the bulk and on the surfaces of CeO_2_. Red, yellow, and dashed spheres indicate O, Ce, and V_O_^••^, respectively. (d, e) Optimized configurations of hole and electron small polarons. Spin density is shown with isosurface levels of 0.01 and 0.002 e/Bohr^3^. (f) Calculated localization energies of holes and electrons in different atomic layers under the surfaces of CeO_2_. For comparison, the intrinsic localization energies of charge carriers in ideal bulk (excluding surface polarization effects) are shown in dashed lines. (g) Formation energies of oxygen vacancies on different surfaces of CeO_2_ compared to those in bulk in the O-rich limit. The shaded area shows the range of possible Fermi energy level positions predicted self-consistently in undoped CeO_2_.

The charge carriers associated with the formation of intrinsic point defects in CeO_2_ are well localized, forming small polarons on cation (electron) and anion (hole) sites (Figure 1d,e). Under common environmental conditions (*T* = 100–1600 K and *P* = 10^–15^–10^2^ atm), the self-consistent Fermi level calculated in bulk CeO_2_ resides at least 2.89 eV above the valence band maximum (VBM) using the BB1K functional, due to the preferential formation of positively charged V_O_. This prediction is consistent with experimental measurements that generally show a Fermi level around 3 eV above the VBM.^32,38^ As the temperature increases, the Fermi level rises away from the VBM. As the Fermi level exceeds 3.4 eV, the charge state transition of V_O_(+2/+1) leads to a reduced concentration of free electrons and more trapped electrons near the V_O_ sites.

### 2.2 Intrinsic Stability of Isolated Ce_Ce_′ and V_O_^••^ in Bulk and on Surfaces

Hybrid QM/MM calculations in bulk CeO_2_ show that V_O_^••^ and V_O_^•^ are the predominant charge states, which are mainly compensated for by free electronic polarons that are not tightly bound to the vacancy site. We therefore further consider the localization site of extra electrons in ceria.

With newly developed hybrid QM/MM models for ceria surfaces (Figure 1b), we calculated the vertical and adiabatic ionization potential (IP) and electron affinity (EA) of several surfaces of CeO_2_ and compared the localization energies of charge carriers into different atomic layers under each surface. Our calculations show that while holes show no preference for localization in any specific atomic layer under a given surface, electrons have a significant tendency to localize on the topmost surface layer (Figure 1f). From the topmost to the third layer beneath the surface, the localization energy of electrons decreases by 0.66, 0.47, and 0.29 eV for the (110), (111), and CeO_4_-t (100) surfaces, respectively. These calculations demonstrate the preference of electron localization itself, independent of the formation of V_O_^••^. This preference is driven by the reduced coordination of surface Ce atoms, which allows greater structural flexibility to accommodate the larger ionic radius of Ce_Ce_′. As quantified by the mass-weighted atomic displacements in Table 2, the extent of structural relaxation follows the order surface > subsurface > bulk, consistent with the trend observed in the calculated electron localization energies. For comparison, within bulk CeO_2_, the binding energy of V_O_^••^ and Ce_Ce_′ in V_O_^•^ is calculated as 0.45 eV, which is comparable to or even lower than the energy favoring surface localization. Moreover, the binding energy of V_O_^•^ and a second associated electron to form V_O_^×^ is calculated as −0.16 eV, indicating that capturing the second charge-compensating electron near the V_O_^••^ site in bulk CeO_2_ is energetically unfavorable. These results imply a distinct preference for charge-compensating Ce_Ce_′ to reside on the topmost surface layers in ceria instead of being trapped near the V_O_^••^ site in bulk.

We further calculated the formation energies of different charge states of V_O_ on the ceria surfaces. These energies are given as a function of the Fermi level position above the VBM in Figure 1g, where *E*_VBM_ is the negative vertical IP of the corresponding surface. We first consider the formation energy of doubly ionized oxygen vacancies, V_O_^••^, showing the intrinsic stability independent of excess electrons (Figure 1c). At a given Fermi level, the formation energies of V_O_^••^ follow the order: (111) > (110) > (100) CeO_4_-t. This trend was found to align with the vertical IPs of surfaces and Madelung potential at surface oxygen sites reported in our previous work,^31^ suggesting the dominant effects from surface electrostatics. The O-terminated (111) surface is quadrupolar and has the highest IP (7.67 eV), corresponding to the most positive Madelung potential on surface oxygen sites. The (110) surface, though intrinsically nonpolar, features oxygen protrusions after relaxation and yields an intermediate IP value (6.11 eV), while the reconstructed polar (100) CeO_4_-t surface has the lowest IP of 5.94 eV. A higher Madelung potential at oxygen sites indicates stronger electrostatic binding, thus increasing the energy cost of oxygen vacancy formation. Our calculated formation energies of isolated V_O_^••^ are consistent with this electrostatic interpretation. Notably, if V_O_^••^ and Ce_Ce_′ are treated as isolated, noninteracting species, the formation of V_O_^••^ on the (111) surface is less favorable than in the bulk, while electron localization remains energetically favorable on the surface, in agreement with predictions from the space-charge model.^12,13,18^

### 2.3 Enhanced V_O_^••^–Ce_Ce_′ Coupling on Ceria Surfaces

When the interaction between V_O_^••^ and Ce_Ce_′ is considered, substantial stabilization of near-surface vacancies is observed. For example, under O-rich conditions, the formation energies of charge-neutral V_O_^×^ (or [V_O_^••^ + 2Ce_Ce_′]) are calculated as 1.95, 1.52, 0.89, and 1.20 eV for the (111)_surface_, (111)_subsurface_, (110)_surface_, and CeO_4_-t (100)_surface_, respectively, which are all significantly lower than that in the bulk (2.91 eV). Moreover, focusing on the most stable CeO_2_(111) surface, the formation energy of V_O_^×^ is lower in the subsurface than in the topmost surface layer, consistent with earlier studies employing periodic supercell models.^9,39^ However, for doubly ionized V_O_^••^, the formation energy difference between subsurface (−4.09 eV at the VBM) and surface (−4.11 eV) is minimal but increases to a 0.43 eV difference after the association with 2 surface electrons (V_O_^×^). Therefore, the preferential formation of subsurface vacancies over surface vacancies on CeO_2_(111) does not arise directly from the V_O_^••^ itself but rather from the additional stabilization gained by coupling with surface electrons. A similar conclusion applies when comparing surface and bulk vacancies, highlighting the importance of considering defect interactions in analysis.

In summary, the enhanced stability of surface vacancies originates from two key factors. Apart from the preferential localization of excess electrons discussed earlier, the enhanced stability also arises from stronger V_O_^••^–Ce_Ce_′ coupling on ceria surfaces. The calculated binding energies of V_O_^••^ and Ce_Ce_′ in V_O_^•^ on the (111), (110), and (100) surfaces are 0.61, 0.56, and 0.84 eV, respectively, which are all higher than 0.45 eV for bulk V_O_^•^ (Table 1), indicating stronger vacancy-polaron coupling on ceria surfaces. Upon association with a second electron, the calculated binding energies between V_O_^•^ and the second Ce_Ce_′ in V_O_^×^ are all positive on the studied surfaces (Table 1), compared to the negative value (−0.16 eV) in the bulk. The enhanced coupling between V_O_^••^ and Ce_Ce_′ on CeO_2_ surfaces than that in bulk can be attributed to easier atomic relaxation on surfaces due to reduced coordination numbers, as manifested in the mass-weighted sum of atomic displacement due to defect formation (Table 2), which is defined as

$${\left(\Delta Q\right)}^{2}=\underset{i}{\sum }m_{i}\Delta {R_{i}}^{2}$$ 1

where *i* are atoms in the active regions, *m*_*i*_ is the atomic weight of the atom *i*, and Δ*R*_*i*_ is the displacement of atom *i* after structural relaxation. A reduced coordination number results in enhanced ionic relaxation upon defect formation, which consequently decreases the required energy for the formation of the defect.

**Table 1:** Binding Energies (eV) between V_O_^••^–V_O_^••^, Ce_Ce_′–Ce_Ce_′, V_O_^••^ and the NN-Site Ce_Ce_′ in V_O_^•^, V_O_^••^ and the NNN-Site Ce_Ce_′ in V_O_^•^, V_O_^•^ and Second-Compensating Ce_Ce_′ in V_O_^×^ in Bulk and on Surfaces of CeO_2_ Calculated at the Dilute Limit Using QM/MM Models^a^

	V_O_^••^–V_O_^•^^•^	Ce_Ce_′–Ce_Ce_′	V_O_^••^–Ce_Ce_′ in V_O_^•^ (NN)	V_O_^••^–Ce_Ce_′ in V_O_^•^ (NNN)	V_O_^•^–2nd Ce_Ce_′ in V_O_^×^
bulk	–1.53	–0.22	0.38	0.45	–0.16
(111) subsurface	–1.15	–0.32	0.53	0.46	1.04
(111) surface	–1.16	–0.32	0.49	0.61	0.44
(110)	–3.25	–0.23	0.56	0.55	0.31
(100) CeO_4_-t	–1.51	–0.62	0.86	0.84	0.29

a Negative values indicate repulsive interactions, while positive values signify attractive interactions.

**Table 2:** Mass-Weighted Sum of Atomic Displacements Δ*Q* (amu^–1/2^ Å) in the Active Regions of QM/MM Models Due to the Formation of V_O_ and Ce_Ce_′, Compared to the Perfect Lattice and Their Coordination Environments

	CN_Ce–O_	Δ*Q* (Ce_Ce_′)	CN_O–Ce_	Δ*Q* (V_O_^••^)	Δ*Q* (V_O_^•^)	Δ*Q* (V_O_^×^)	Δ*Q* (V_O_′)	Δ*Q* (V_O_″)
bulk	8	1.44	4	5.66	5.78	5.70		
(111) subsurface	8	1.79	4	6.23	6.64	7.21	8.16	8.66
(111) surface	7	2.90	3	7.37	7.99	8.79	9.48	10.21
(110)	6	3.43	3	8.60	9.11	9.31	10.14	10.73
(100) CeO_4_-t	4	3.24	2	7.64	6.91	8.08	9.22	9.55

As a result, at a given Fermi level, near-surface V_O_^••^ tends to trap more electrons than bulk counterparts, as shown by the relative stability of their charge states associated with varying numbers of Ce_Ce_′ in Figure 1g. For example, as the Fermi level rises 3 eV above the VBM, the V_O_ in the bulk does not trap electrons and stabilizes in the +2 charge state, while the most stable charge states of V_O_ on the (111)_surface_, (111)_subsurface_, and (110)_surface_, are +1 (one trapped Ce_Ce_′), 0 (two trapped Ce_Ce_′), and 0, respectively. As *E*_f_–*E*_VBM_ increases to 3.5 eV, where V_O_^•^ becomes the most stable defect in bulk CeO_2_, V_O_′, which is associated with three trapped Ce_Ce_′, becomes dominant on the (111) and (110) surfaces.

Given that the Fermi level in CeO_2_ is typically pinned near the polaronic Ce 4f band as we calculated self-consistently (*E*_f_–*E*_VBM_ > 2.9 eV) and confirmed in experiment,^32,38,40^ we propose a defect heterogeneity in ceria, differing from predictions from conventional periodic supercell models and simple space-charge models. While the formation of a V_O_^••^ in the bulk requires the compensation of two Ce_Ce_′ to maintain charge neutrality, the two Ce_Ce_′ are not likely to be located near the V_O_^••^ site but tend to segregate on ceria surfaces. As a result, the ratio of their quantities in an undoped ceria system, *n*_Ce_Ce_^′^_/*n*_V_O_^••^_, is expected to decrease from the surface (>2) to the bulk (<2), rather than maintaining a constant ratio of 2 across the whole system, as a result of the preferential localization of electrons and enhanced vacancy-polaron coupling on surfaces.

### 2.4 Experimental Evidence of Preferential Electron Localization on Surfaces of Ceria Nanoparticles

To test our proposed defect heterogeneity model, we combined experiment and large-scale atomistic simulations to characterize the defect distribution in ceria nanoparticles. Octanoic-acid-modified ceria nanoparticles were synthesized by a continuous flow hydrothermal method, as reported previously.^41^ The resulting particles were homogeneous with a narrow size distribution and remained well-crystallized even in the range of ultrasmall size, as observed by STEM (Figure 2a–f).

**Figure 2:**
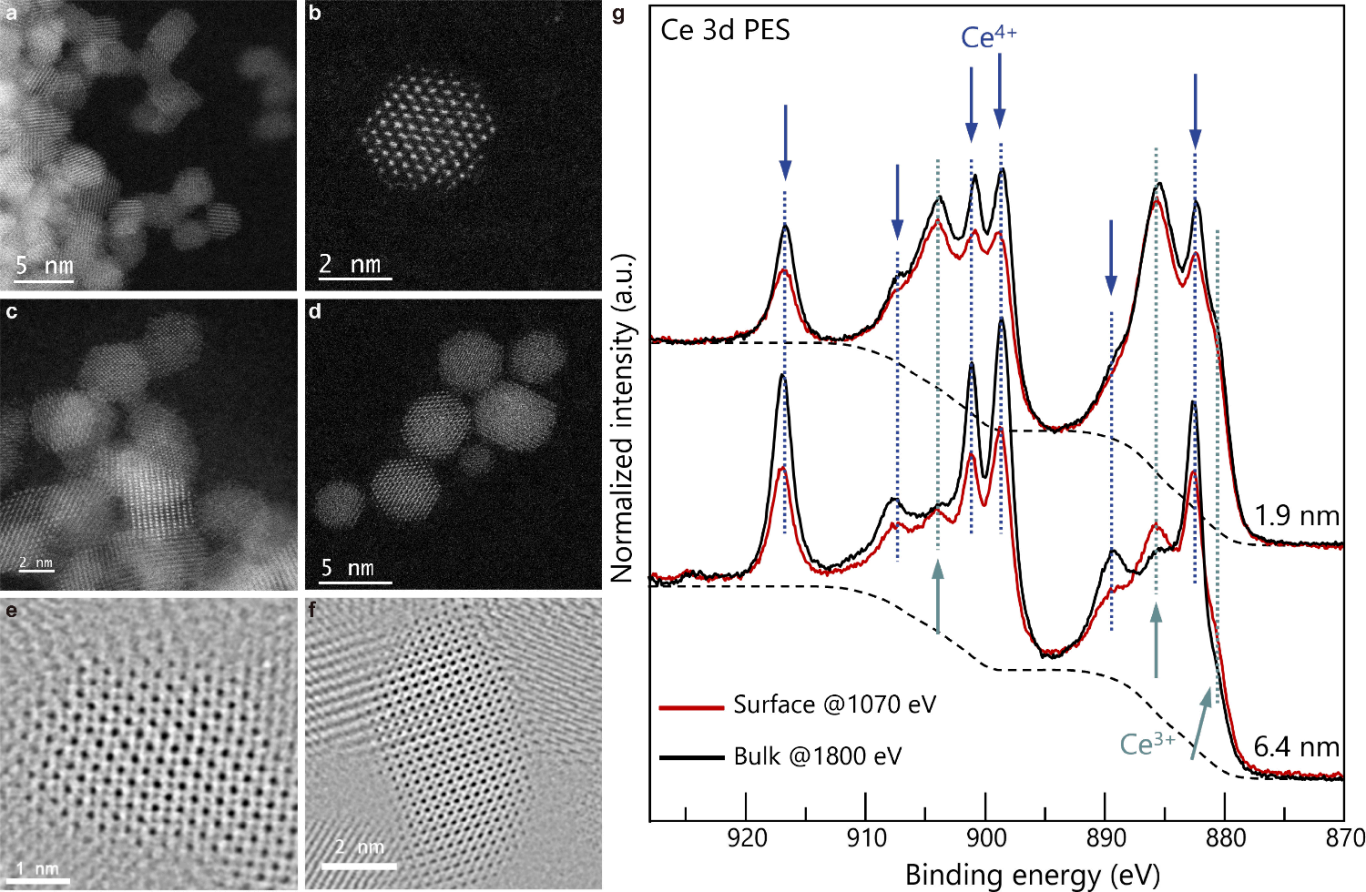
Experimental characterization of ceria nanoparticles. (a–f) STEM images of ceria nanoparticles (3.3 nm) using (a, b, c, d) annular dark-field and (e, f) annular bright-field modes. (g) Synchrotron XPS measurements for Ce 3d from organic-modified ceria nanoparticles of two different sizes (1.9 and 6.4 nm) were conducted using incident X-ray energies of 1070 and 1800 eV for the surface and bulk chemical states, respectively. The background function was determined using the Shirley algorithm. The normalization was performed such that the pre-edge intensity was set to 0 and the postedge intensity to 1.

The chemical state of organically modified ceria was intensively studied. In our previous work, TEM-electron energy loss spectroscopy (EELS) analysis was employed to probe the chemical state of Ce by analyzing the Ce M_4,5_ edge for each atomic layer of the particles.^42–44^ For larger particles (∼10 nm), Ce^3+^ was observed only at the surface. However, as the particle size decreased to below 7 nm, Ce^3+^ was also observed inside the nanoparticles because of the significant lattice distortion caused by nanosizing. While this technique provides a clear image of the chemical state distribution, its limitation lies in its focus on a single particle rather than the whole sample.

In this study, X-ray photoelectron spectroscopy (XPS) was employed to obtain the bulk and surface information on the collective nanoparticles. For depth analysis of the samples, synchrotron X-ray techniques were used, and photoelectron spectra were obtained by changing the energy of the incident X-rays. Figure 2g shows the synchrotron XPS data for Ce 3d spectra of two representative organic modified CeO_2_ nanoparticles with different sizes (1.9 and 6.4 nm), using two different incident X-rays (1070 and 1800 eV). The 1070 eV X-rays provided surface-sensitive information (∼1 nm), while the 1800 eV X-rays probed the bulk chemical states (∼2 nm), as shown in the inelastic mean free path (IMFP) and simulated probing lengths in Figure S3. As a result, the differences in Ce chemical states were observed between the two samples: the 1.9 nm particles had a higher Ce^3+^ content, whereas more Ce^4+^ were observed for larger particles; this is mainly because of the disordering of O as discussed previously.^41^ When the surface and inside of the same particles were compared, larger amounts of Ce^3+^ were detected at the surface. The particle size of 6.4 nm is slightly larger than the two probing lengths, and the chemical states of different depths are clearly shown. By fitting the XPS peaks, the intensity fractions of Ce^3+^ peaks of 6.4 nm CeO_2_ were found to be 39.3% for 1 nm probing length (1070 eV X-rays) and 34.2% for 2 nm probing length (1800 eV X-rays). On the other hand, larger intensity fractions of Ce^3+^ peaks were observed for 1.9 nm with different X-ray energies (64.9% for 1 nm probing length (1070 eV X-rays) and 48.3% for 2 nm probing length (1800 eV X-rays)). All the fitting results for each data are shown in the Supporting Information (Table S2 and Figure S4).

The synchrotron XPS data, together with previous TEM-EELS results,^42,44^ clarified the spatial distribution of the Ce chemical states in the particles, confirming the preferential localization and segregation of Ce^3+^ electrons on ceria surfaces, as predicted by the hybrid QM/MM embedded-cluster models. A similar tendency was verified with the total information on the collective particles. Furthermore, a consistent trend has also been shown in our EELS investigation of Cr^3+^-doped ceria,^43^ wherein Cr was shown to substitute for Ce sites. Reassessment of the EELS data reveals an increased Cr/Ce ratio at the surface relative to the bulk, similar to the localization tendency of Ce^3+^.

### 2.5 Large-Scale Monte Carlo Simulations of Defect Distribution in Ceria Nanoparticles

For further confirmation of our predictions and to clarify the relative positions of V_O_^••^ and Ce^3+^, we employed large-scale unbiased Monte Carlo simulations with interatomic potential techniques to investigate the stable defect distribution in the reduced ceria nanoparticles. The detailed workflow is shown in Figure 3a. We constructed three different Wulff-construction-like morphologies: an octahedral-shaped model exclusively terminated by the most stable (111) surfaces, a truncated octahedral model with mixed termination by the three main low-index surfaces, and a nanocube model terminated by the O-t (100) surfaces. All these morphologies can be controllably synthesized in experiment and play distinct roles in catalytic reactions.^45,46^

**Figure 3:**
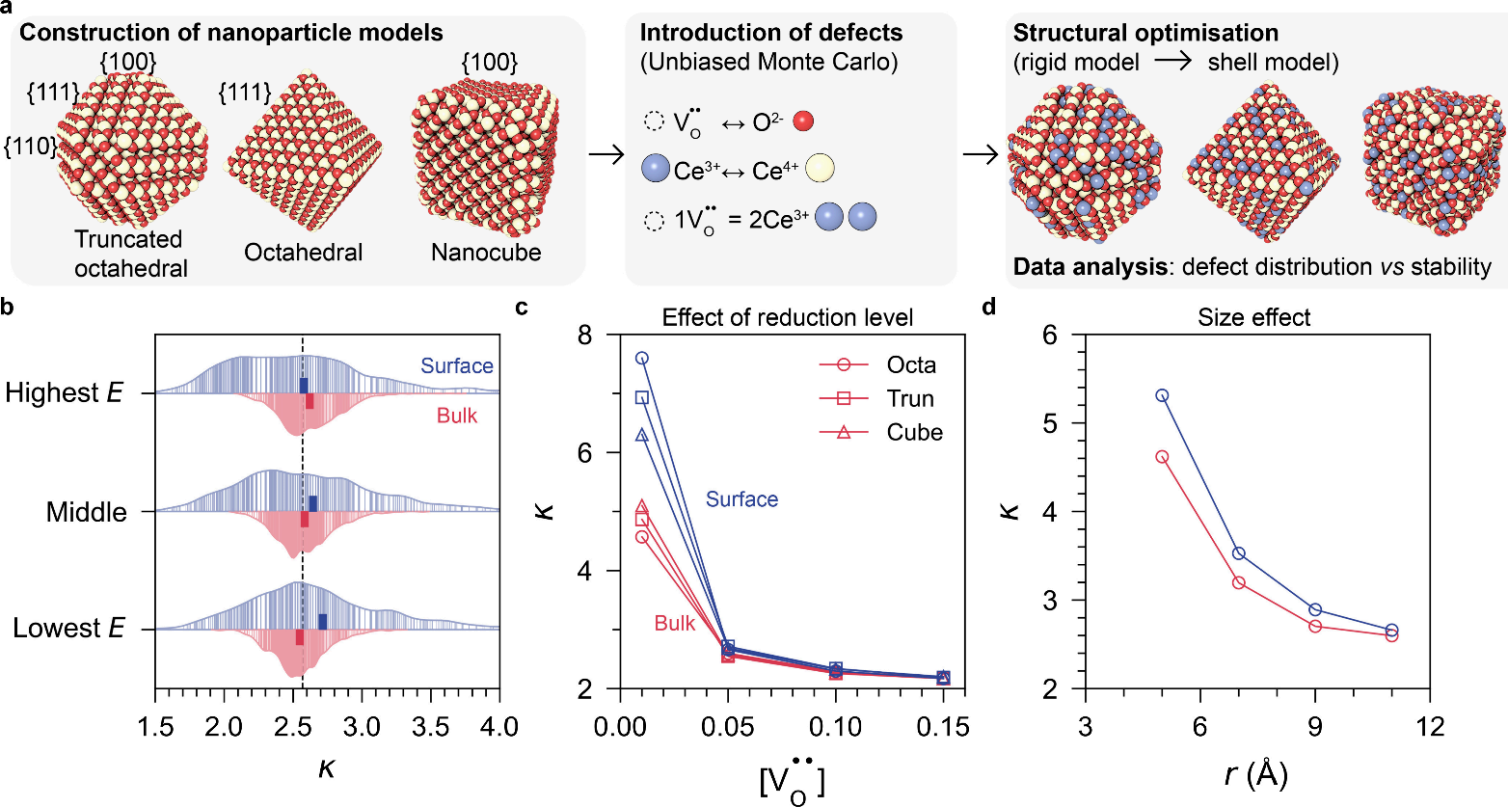
Distribution of V_O_^••^ and Ce_Ce_′ in ceria nanoparticles and its relationship with stability assessed by unbiased Monte Carlo simulations. (a) Simulation workflow. (b) Statistical violin plot shows a representative picture of defect distribution (κ = [Ce^3+^]/2­[V_O_^••^]) in bulk and on the surface, calculated based on an intrinsically nonstoichiometric octahedral nanoparticle model with an additional 5% V_O_^••^. Results are categorized into the highest, medium, and lowest energy groups, each containing 1,000 configurations out of the optimized 10,000 total configurations. The dark bars represent the mean values for each group. (c) κ ratio in bulk and on the surface as a function of the concentration of V_O_^••^. (e) κ ratio in bulk and on the surface as a function of particle radius in octahedral-shaped nanoparticles of varying sizes. (c, d) Results were averaged to only include the 1000 configurations with the lowest energies from each simulation.

The unbiased Monte Carlo simulations were initiated by randomly introducing fixed numbers of V_O_^••^ and Ce^3+^ into the lattice of ceria, as implemented in the Knowledge-Led Master Code (KLMC) code and KLMC-GULP task-farming interface.^47–49^ V_O_^••^ is allowed to exchange with O^2–^, while Ce^3+^ is allowed to exchange with Ce^4+^ by using the Monte Carlo algorithm. To maintain charge neutrality, the total ratio of newly introduced defects, *n*_Ce^3+^_/*n*_V_O_^••^_, was fixed at 2:1. For each stoichiometry, we generated 10,000 random initial structures with random distributions of V_O_^••^ and Ce^3+^, which were then fully optimized using a three-step process from rigid- to shell-model interatomic potentials. Details are given in the Methodology section. With this strategy, we were able to investigate how the defect distribution affects the nanoparticle stability under a given defect concentration and determine the thermodynamically stable defect distribution in ceria nanoparticles. After the structural optimization of each configuration, we calculated the concentration ratio of Ce^3+^ and V_O_^••^ in the bulk and surface regions, respectively, which is defined as

$$\kappa =\frac{\left[{\mathrm{Ce}}^{3+}\right]}{2\left[V_{O}^{••}\right]}=\frac{n_{{\mathrm{Ce}}^{3+}}/\left(n_{{\mathrm{Ce}}^{3+}}+n_{{\mathrm{Ce}}^{4+}}\right)}{2n_{V_{O}^{••}}/\left(n_{V_{O}^{••}}+n_{O^{2-}}\right)}$$ 2

Physically, κ represents the average number of electrons compensating for each V_O_^••^ in the region. In an ideal homogeneous system of bulk reduced ceria, κ = 2, corresponding to complete local charge compensation by two Ce^3+^ ions per V_O_^••^. In ceria nanoparticles, however, it has been shown experimentally that intrinsic Ce^3+^ species can form in the absence of oxygen vacancies, driven by significant lattice distortions at ultrasmall sizes.^41^ We reproduced this characteristic using intrinsically nonstoichiometric nanoparticle models that retain the surface structure of CeO_2_ and incorporate additional Ce^3+^ to preserve the overall charge neutrality. As a result, in such systems, the overall κ exceeds 2 after reduction (see Methodology for details). Here, κ is evaluated locally between the bulk and surface regions. First, we performed unbiased Monte Carlo simulations to check the preferential positions of intrinsic Ce^3+^ in the absence of additional oxygen vacancies. In all three morphologies, the most stable configurations show complete localization of Ce^3+^ at surface sites rather than in the bulk, consistent with experimental observations of large nanoparticles over 10 nm.^42^ We further introduced additional V_O_^••^ and charge-compensating Ce^3+^ into the nanoparticles. To minimize the effects of randomness and ensure statistical significance, for each stoichiometry, we selected three groups of 1000 configurations with the lowest, median, and highest energies from the pool of the optimized and ranked 10,000 samples and checked the defect distribution. As an example, Figure 3b shows a statistical violin plot of the three groups obtained in an octahedral-shaped nanoparticle with the addition of 5% V_O_^••^. In the most stable group of 1000 configurations, the κ ratio is higher at the surface than in the bulk. In contrast, the least stable group exhibits the opposite trend, while the intermediate group shows a comparable average κ ratio in both the bulk and surface regions. This comparison indicates that the accumulation of electronic polarons on surfaces enhances the overall stability of the ceria nanoparticles. This simulation agrees well with our QM/MM predictions based on defect formation energies, showing that electron polarons have a strong preference to localize on the surface in reduced ceria, rather than being trapped in bulk.

In the following analyses, only the 1000 most stable configurations from the 10,000 initial structures were considered. Figure 3c shows the averaged κ values in ceria nanoparticles with different morphologies and under different levels of reduction. With the increase in the degree of reduction, we observe that electrons have less preference to localize on the surface across all studied systems. Moreover, with a fixed [V_O_^••^] of 5% in octahedral nanoparticle models, we studied the size effects. As shown in Figure 3d, with increasing particle size, the overall κ decreases, and the difference between surface and bulk κ values becomes less pronounced, indicating a weakened spatial heterogeneity in defect distribution with increasing particle size. For ultrasmall nanoparticles, the surface can have a very high concentration of Ce_Ce_′, attributable to their nanomorphology and large lattice distortion, despite a low [V_O_^••^], which agrees well with experiment.^41,42^

The measured extent of surface segregation of Ce^3+^ in the experiment is higher than the predicted equilibrium-state distribution from Monte Carlo simulations (Tables S3 and S4). XPS measurements show Ce^3+^ surface-to-bulk ratios of 64.9 vs 48.3% for 1.9 nm nanoparticles and 39.3 vs 34.2% for 6.4 nm particles. Monte Carlo simulations predict much smaller surface–bulk differences in Ce^3+^ concentrations (typically ∼ 1.0%). This discrepancy may arise from kinetic factors: under reducing conditions, oxygen removal preferentially initiates at surfaces, while anion extraction from the bulk requires long-range diffusion and overcoming associated energy barriers. Furthermore, a transition in the preferential localization of electrons is observed in the (111)-terminated octahedral model. When the concentration of V_O_^••^ exceeds 5%, simulations show that the Ce^3+^ population of surface sites becomes lower than that of bulk sites. Comparative analysis of the binding energies of V_O_^••^–V_O_^••^ and Ce^3+^–Ce^3+^ pairs from QM/MM calculations (Table 1) reveals that the repulsive interactions between these species are stronger at the (111) surface than in the bulk, indicating a reversed preference for bulk segregation, as seen in the Monte Carlo results in Table S3. By contrast, for (110) surfaces, QM/MM calculations show enhanced V_O_^••^–V_O_^••^ repulsion but reduced Ce^3+^–Ce^3+^ repulsion, resulting in higher κ values on the surface across all studied reduction levels, as seen in truncated octahedral nanoparticles. These findings show that variations in defect–defect interactions across different ceria surfaces can give rise to distinct surface chemistry and further demonstrate the pronounced dependence of ceria nanoparticle properties on their shape and size.

Figure 4a–c shows the atomic structures of the most stable configurations of reduced nanoparticles with truncated octahedral shapes. Initially, upon reduction, both V_O_^••^ and Ce^3+^ prefer isolated positions. With an increasing degree of reduction, the atomic arrangements in nanoparticles become progressively more disordered. In addition, a higher number of V_O_^••^–Ce^3+^ pairs occur in the NN positions, as highlighted by the bonds in the snapshots. Figure 4d–k shows statistics of the relative positions of defects in ceria nanoparticles under varying degrees of reduction. The average separation among defect types, including V_O_^••^ with its first- and second-nearest Ce^3+^, V_O_^••^–V_O_^••^, and Ce^3+^–Ce^3+^, decreases significantly as the reduction level increases.

**Figure 4:**
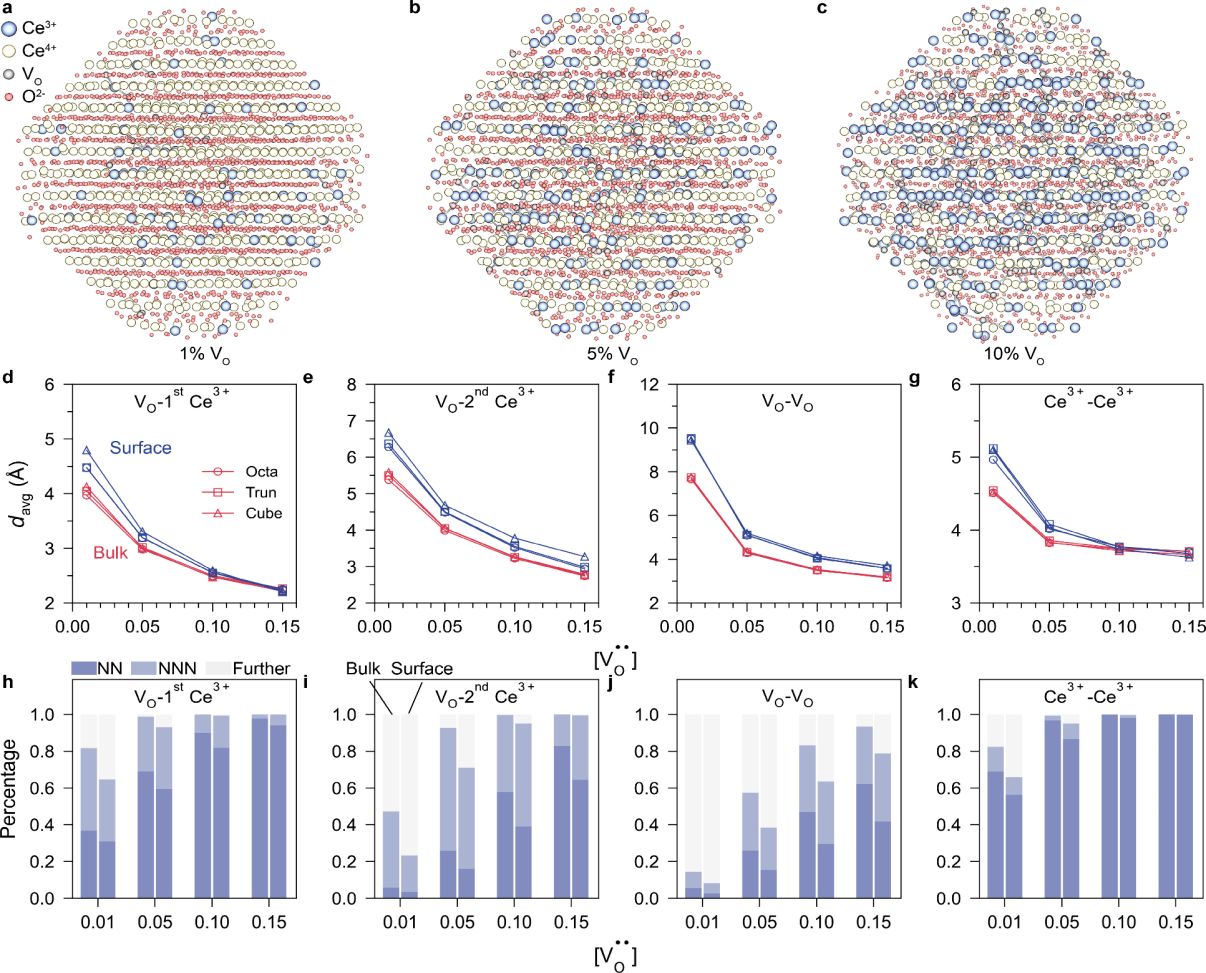
Defect distribution in ceria nanoparticles at different extents of reduction. (a–c) Snapshots of the most stable configurations of reduced nanoparticles with truncated octahedral shapes obtained from unbiased Monte Carlo simulations. Bonds between V_O_^••^–Ce^3+^pairs were shown to highlight the relative positions. (d–k) Relative positions of defects in reduced nanoparticles, including interactions between V_O_^••^ and its first- and second-nearest Ce^3+^, V_O_^••^–V_O_^••^ pairs, and Ce^3+^–Ce^3+^ pairs. (d–g) Average distances among these defect pairs. (h–k) Percentages of these defect pairs in the nearest (NN), next-nearest (NNN), and further neighboring configurations, based on the truncated octahedral models. The stacked columns on the left and right within each stoichiometry represent the bulk and surface regions, respectively.

Our unbiased Monte Carlo simulations supported several conclusions drawn from QM/MM predictions and experiment. First, the proposed defect heterogeneity is evident in slightly reduced ceria, whereas in highly reduced ceria the defect distribution becomes more homogeneous in bulk and surface regions (Figure 3c,d). The repulsive interactions between the same species (Table 1), as well as the association between V_O_^••^ and Ce^3+^, govern the overall defect distribution in highly reduced ceria. As also shown in the stress-inclusive space-charge model of Sheldon and Shenoy,^18^ the compressive strain in the surface region associated with the larger ionic radius of Ce^3+^ acts to counter its surface segregation and the depletion of V_O_^••^, in agreement with our results, particularly on the reversed defect segregation preference seen in highly reduced octahedral nanoparticles. Second, in all studied nonstoichiometric nanoparticles, the distance between a V_O_^••^ and its second-nearest Ce^3+^ is much greater compared to that of the nearest Ce^3+^ (Figure 4d,e,h,i). At the dilute limit in bulk CeO_2_, our QM/MM calculations also showed that the localization of the second charge-compensating electron near the V_O_^••^ sites is energetically unfavorable in bulk and less favorable on surfaces (Table 1). Third, for the association of V_O_^••^ and Ce^3+^, QM/MM calculations predict that the NNN configuration is slightly more stable than the NN configuration (Table 1). In the nanoparticle simulations of low levels of reduction (*x* < 0.05), the second electron tends to form a free polaron, located far away from the V_O_^••^ sites. With an increasing degree of reduction, the NNN site becomes the predominant position for the localization, persisting until a very high defect concentration is reached (Figure 4i). This finding is also supported by previous periodic DFT calculations and experimental STM images from Sauer et al.,^10,16^ who concluded that at least one of the excess electrons localizes not directly adjacent to the V_O_^••^ site. Only under a very high defect concentration ([V_O_^••^] > 0.10) does the second compensating Ce^3+^ have to occupy the NN site to minimize the repulsion with other Ce^3+^. Finally, the interaction between Ce^3+^ is much less repulsive than the V_O_^••^–V_O_^••^ interaction due to their lower effective charges (Table 1). Our QM/MM calculations show that the binding energies between two Ce^3+^ ions at the first, second, and third nearest-neighbor positions are −0.22, −0.32, and −0.30 eV, respectively. As a result, despite the repulsive interaction, the relative magnitudes suggest a thermodynamic preference for Ce^3+^ to reside in close proximity when it is not separated over long distances, which is also seen from simulations of nanoparticles (Figure 4g,k).

## 3 Discussion and Conclusions

Oxygen vacancies and electron polarons in easily reducible metal oxides, such as CeO_2_, TiO_2_, and WO_3_, have a strong effect on their transport and catalytic properties. Understanding the formation, interaction, and distribution of defects is critically important but remains incompletely understood. The space-charge model predicts a positive electrostatic potential near the surface region, which favors the accumulation of electrons and depletion of V_O_^••^.^13,18^ These models treat V_O_^••^ and Ce^3+^ as independent species. In contrast, prior periodic DFT supercell calculations have generally focused on the charge-neutral case, V_O_^×^ or [V_O_^••^ + 2Ce^3+^], which predicted that surface vacancies have lower formation energies compared to those in the bulk.^9,16^ These calculations inherently treat V_O_^••^ and Ce^3+^ as a coupled defect complex.

Our present work employs hybrid QM/MM embedded-cluster models with explicit electrostatic embedding environments and long-range polarization. This approach allows us to consider the formation of V_O_^••^ and Ce^3+^ both independently and in interaction, enabling the examination of all accessible charge states in the bulk and at surfaces of CeO_2_. Our results show that, with normal Fermi levels pinned near the polaronic Ce 4f band around 3 eV above the VBM, bulk oxygen vacancies typically retain ionized states, trapping no or at most one electron, whereas surface vacancies can readily accommodate two or more electrons. The origin of this charge state difference is the strong tendency for electron localization at ceria surfaces, which exceeds the relatively weak electron binding associated with bulk V_O_^••^. As a result, although the formation energies of V_O_^••^ are higher on the most stable CeO_2_(111) surface than those in the bulk, consistent with the prediction by the space-charge models,^13^ coupling with localized surface electrons significantly lowers the overall formation energy of the defect complexes, as shown in previous DFT studies.^9,16^ Therefore, the ease of forming oxygen vacancies at ceria surfaces is primarily driven by the favorable localization of excess electrons and enhanced coupling of V_O_^••^ and Ce^3+^ in the surface region. Based on these results, we propose a spatially heterogeneous defect distribution in ceria, where the surface region is expected to have higher Ce^3+^/V_O_^••^ ratios than the bulk.

To bridge the gap between the dilute models in QM/MM calculations and real systems with higher concentrations of interacting defects, we conducted synchrotron XPS characterization and large-scale unbiased Monte Carlo simulations on ceria nanoparticles, which confirmed our predictions and further showed the effects of nanoparticle size and reduction levels. At higher defect concentrations, repulsive interactions between defects of the same type and defect-induced stress become increasingly important, resulting in a less significant extent of defect heterogeneity or even reversed location preference between bulk and surfaces, which agrees with the prediction by the stress-inclusive space charge model.^18^ Our combined QM/MM and Monte Carlo framework captures both the localized behavior of individual defect species and their concentration-dependent interactions, thereby providing a more realistic description of defect chemistry in reduced ceria under equilibrium conditions. A limitation of the present study is that it focuses solely on intrinsic defects in CeO_2–*x*_ and does not consider the effects of extrinsic impurities or environment-induced surface species. Water is ubiquitous in ambient^50^ and catalytic environments, which dissociates easily on the ceria surfaces, and can refill oxygen vacancies,^51^ resulting in the formation of surface hydroxyls, protons, or hydride species.^52–54^ The presence of these species may significantly modify defect energetics and charge equilibria, especially in nanoscale ceria, where surface effects dominate.

Our findings regarding the preferential localization of electrons on surfaces offer new insights into the versatile applications of ceria in technological applications, including heterogeneous catalysis and ionic conduction. The electron-rich surfaces of ceria facilitate the interaction and stabilization of metal clusters^55,56^ and enhance the activation of molecules^57^ through charge transfer, explaining the high catalytic efficiency in various reactions. Furthermore, in the electronic-poor bulk region, oxygen vacancies can migrate freely in the ceria lattice without the spatial barrier of large Ce^3+^ ions.^11^ This enhanced mobility of oxygen vacancies is advantageous for applications such as solid oxide fuel cells (SOFCs) and oxygen carriers in chemical looping reactions, where efficient ionic conduction is essential. Moreover, the unusual electron distribution in nanoparticles may enable new applications in electronic devices such as a single-electron transistor.^58^ The computational approaches presented here could be powerful to understand the new properties of ultrasmall metal oxides caused by the heterogeneous distributions of defects and electrons.

## 4 Methodology

This work integrates several computational approaches, including interatomic potential techniques, hybrid QM/MM embedded-cluster approaches, and unbiased Monte Carlo simulations, with experimental synthesis of ceria nanoparticles and characterization using STEM and synchrotron XPS.

### 4.1 Hybrid QM/MM Embedded-Cluster Approaches

The electrostatic Coulomb interactions and long-range polarization are explicitly included in all computational models employed in this study. The representation of ionic oxides relies on the shell-model interatomic potential,^7,59,60^ in which each ion is represented by a core and a shell, both carrying partial charges whose sum equals the formal ionic charge. The displacement of shells relative to their cores models the polarization response to external fields arising from all other ions and external lattice discontinuities such as defects and surfaces. In the hybrid QM/MM model, these electrostatic interactions are calculated explicitly in the MM regions and are transferred to the QM region via the electrostatic embedding scheme, while the defects included in the QM region in turn polarize the heterogeneous environment.^61^ In this work, the Python-based version of the ChemShell package,^30,61,62^ which links NWChem^63,64^ as the QM driver and GULP^65,66^ as the MM driver, with DL-FIND^67^ as the structural optimization module, was employed in QM/MM calculations.

The computational setup for defect calculations in bulk CeO_2_ is in alignment with our previous work.^7^ For QM calculations, we employed the hybrid meta-GGA functional BB1K^33^ to account accurately for localized charge carriers in ceria in the QM region. We employ the Def2-TZVP basis set^68^ for O ions and a [4s4p2d3f] basis set^69,70^ with the Stuttgart-Dresden quasi-relativistic effective core potential^71^ for Ce ions. Our developed shell-model interatomic potential^7^ was used in the MM regions to consider the long-range polarization and atomic displacement induced by charged defects. The QM/MM interface region comprises a layer of cations surrounding the QM cluster, which is described by specially designed local pseudopotentials. The pseudopotentials are constructed as a linear combination of three Gaussian functions and were optimized to minimize the residual forces on all atoms in the active region (QM, interface, and MM-active) as well as the scatter of deep core levels. More details can be found in our previous work.^7^ In this study, we examined all possible charge states of defects and low-spin configurations of holes, some of which were not considered in our previous work.

We developed hybrid QM/MM models to study the properties of oxygen vacancies and electronic polarons on the ceria surfaces. First, periodic slab models with a fixed bottom region were cleaved from the unit cell of CeO_2_ and optimized to obtain the relaxed surface structures under the description of interatomic potentials. Then, hemispherical QM/MM models were constructed based on optimized surface slabs, with 81, 93, and 86 QM atoms for the (111), (110), and CeO_4_-t (100) surfaces, respectively. Cut-off radii of 15 and 30 Å were employed to divide the model into active- and frozen-MM regions, respectively, consistent with bulk models. This partition yields ∼700 active atoms for explicit structural optimization, accounting for the large polarization field and displaced atoms induced by defect formation.

In the energy calculations for charged systems, the Jost correction^72,73^ is employed to compensate for the long-range polarization external to the finite-size relaxed region. The formula of this correction varies between bulk and surface models:

$$E_{\mathrm{Jost}}^{\mathrm{bulk}}=-\frac{Q^{2}}{2R}\left(1-\frac{1}{\varepsilon }\right)$$ 3

$$E_{\mathrm{Jost}}^{\mathrm{surface}}=-\frac{Q^{2}}{2R}\left(\frac{\varepsilon -1}{\varepsilon +1}\right)$$ 4

where *Q* is the net charge of the system, *R* is the radius of the active region, and ε_0_ (24.5) and ε_∞_ (5.31)^7^ are the static and high-frequency dielectric constants of CeO_2_, which are used for adiabatic and vertical processes, respectively.

Vertical and adiabatic ionization potentials (IPs) and electron affinities (EAs) were calculated by

$$\mathrm{IP}=\left(E_{+1}+E_{\mathrm{Jost}}\right)-E_{0}$$ 5

$$\mathrm{EA}=E_{0}-\left(E_{-1}+E_{\mathrm{Jost}}\right)$$ 6

where *E*_0_, *E*_+1_, and *E*_–1_ are the calculated QM/MM energies of the charge-neutral, *q* = +1, and *q* = −1 systems, respectively. For vertical calculations, relaxation is allowed only at the electronic (and MM shell) level, with the ionic (and MM core) positions remaining fixed at the *E*_0_ state. Adiabatic processes further include ionic relaxations, resulting in a localized hole or an electron in the optimized systems with +1 and −1 charge states, respectively.

The formation energy *E*_f_[*X* ^*q*^] of a charge defect *X* in the charge state *q* is calculated by

$$E_{f}[X^{q}]=E[X^{q}]-E_{0}-\sum_{i}n_{i}\mu_{i}+qE_{F}+E_{\mathrm{Jost}}$$ 7

where *n*_*i*_ represents the number of species added (>0) or removed (<0) from the system, μ_*i*_ is the chemical potential of species *i*, and *E*_F_ is the Fermi energy relative to the VBM. Note that the absolute positions of the VBM differ across bulk and different surface models; therefore, the respective VBM positions are employed in defect calculations. The limits for O-rich (μ_O_ = 0 eV and μ_Ce_ = −11.28 eV) and O-poor (μ_O_ = −3.94 eV and μ_Ce_ = −3.40 eV) conditions are determined by the experimental formation enthalpy of CeO_2_ (−11.28 eV) and the competing phase Ce_2_O_3_ (−18.62 eV).^74^ The QM energies of the reference states were calculated using consistent basis sets and functionals. For oxygen defects, the QM energy of *O*_2_(g) is used as the reference state. The energy of Ce(s) is used as the reference for cerium defects, which is derived by subtracting the sum of the four ionization energies (73.745 eV^74^) and the sublimation enthalpy (4.380 eV^74^) of Ce from the QM energy of Ce^4+^(g). Details were explained in our previous work.^7^

Equilibrium-state defect concentration at a given temperature *T* is calculated by

$$[X^{q}]=N_{x}g_{x^{q}}\exp\left(-\frac{E_{f}[X^{q}]}{k_{B}T}\right)$$ 8

where *N*_*x*_ is the density of defect sites, *g*_*x* ^*q*^_ is the degeneracy of the charged defect, and *k*_B_ is Boltzmann’s constant. The self-consistent Fermi level and carrier concentrations are determined according to the density of states (DOS) of the pristine system and the charge neutrality constraint, as implemented in the SC-FERMI code.^34^ The DOS of CeO_2_ was calculated using the Vienna Ab-initio Simulation Package (VASP)^75^ code with the PBE0 ^76^ hybrid functional, and the positions of unoccupied states were shifted to reproduce the experimental band gap of 4 eV.^40^ This band gap value was determined by a recent study combining steady-state and ultrafast transient absorption spectra, which identified the true onset of the O 2p → Ce 4f transition at 4.0 eV. This contrasts with earlier reports of 3.0–3.6 eV based on Tauc analysis, which was reinterpreted as excitations into localized polaronic 4f states, incorporating the self-trapping (localization) energy of electrons.

Oxygen partial pressure *p*_O_2__ in the growth environment is directly correlated to the chemical potential of oxygen, thereby affecting defect formation energies in metal oxides. The chemical potential as a given *T* and *p*_O_2__ is calculated by^7,77^

$$\mu_{O}\left(T,p_{O_{2}}\right)=\mu_{O}\left(T,p_{O_{2}}^{0}\right)+\frac{1}{2}k_{B}T\ln\;\frac{p_{O_{2}}}{p_{O_{2}}^{0}}$$ 9

where *p*_O_2__^0^= 1 atm is the reference zero state, under which the chemical potential μ_O_(0 K, *p*_O_2__^0^) is defined as $\frac{1}{2}E_{O_{2}\left(g\right)}$ and rescaled to 0 eV. The chemical potential at elevated temperatures can be derived from experimental data^78^ of the enthalpy *H* and entropy *S* of *O*_2_(*g*) according to

$$\mu_{O}\left(T,p_{O_{2}}^{0}\right)=\frac{1}{2}\left[H\left(T,p_{O_{2}}^{0}\right)-H\left(0\;K,p_{O_{2}}^{0}\right)\right]-\frac{1}{2}T\left[S\left(T,p_{O_{2}}^{0}\right)-S\left(0\;K,p_{O_{2}}^{0}\right)\right]$$ 10

In metal oxides, the ionization potential (IP) and electron affinity (EA) are surface-dependent quantities due to the difference in electrostatic potentials at ionic sites under different surface terminations.^31,32^ To determine the localization energies of electrons and holes, we first calculated the IP and EA of several surfaces of CeO_2_, which represent the minimal energies required to add or remove an electron without allowing any change in the positions of the ions, respectively (vertical processes). We then introduce electrons and holes into different atomic layers under each surface, where atoms are allowed to relax fully to their new equilibrium positions (adiabatic processes). The energy difference between the adiabatic and vertical processes corresponds to the localization energy. While the hole in the bulk is primarily localized on oxygen sites in the stationary calculation (with a spin density of 0.82), it does not fulfill the Mott criterion for self-trapping, *E*_loc_ > 1/2 *W*, where *W* is the bandwidth of oxides. Given that the O 2p band in CeO_2_ is relatively broad (∼4.5 eV), holes in bulk CeO_2_ with a localization energy of 1.6 eV are unable to remain stationary and would not be observed as a self-trapped polaron.^79^ However, holes at the surface, which have substantially higher localization energies, especially on CeO_2_(100), might be stabilized and observed as self-trapped species.

### 4.2 Unbiased Monte Carlo Simulations

We conducted unbiased Monte Carlo simulations by integrating the KLMC^47,48^ code for random structural generation with GULP^65,66^ for interatomic-potential-based lattice energy calculations and structural optimization. To improve computational efficiency, a recently developed task-farming KLMC-GULP interface^49^ that allocates CPU cores into various numbers of work groups is used to launch successive GULP calculations and support massively parallel calculations on high-performance computing (HPC) platforms.

We constructed three models of ceria nanoparticles with different morphologies, as shown in Figure 3a. To preserve charge neutrality in initial configurations, we substitute several Ce^4+^ with Ce^3+^ undercoordinated corner and edge sites to maintain charge neutrality, which is termed “intrinsically nonstoichiometric” models. Random distribution of V_O_^••^ and Ce^3+^ was introduced in nanoparticles using the Monte Carlo algorithm, which allows the random exchange of V_O_^••^ with O^2–^, and Ce^3+^ with Ce^4+^. Different stoichiometries were generated while keeping a ratio of 2:1 for randomly introduced V_O_^••^ and Ce^3+^, preserving the overall charge neutrality. In each Monte Carlo simulation, 10,000 random configurations were generated.

The randomly generated configurations underwent a three-stage structural optimization process. A recently developed set of shell-model potentials for reduced ceria^32^ was used. First, the rigid model interatomic potential was employed for structural optimization; i.e., each ion is only described by a core with its formal charge, and the short-range potentials remain the same as the shell model. While electronic polarization is omitted, rigid model potentials significantly improve the computational efficiency to approach the minima in structural optimization. Then, shell-model potentials were used for a single-point shell relaxation, allowing ionic polarization to be included in rigid-model-optimized structures. Finally, ionic relaxation is allowed to fully optimize the coordinates of cores and shells.

Data analysis focuses on how the distribution of V_O_^••^ and Ce^3+^ affects the stability of the reduced nanoparticles. We classified the “bulk” and “surface” regions of nanoparticles by counting the coordination numbers of ions in initial models, where the undercoordinated ions are included in the surface region. After partition, the ions in each region may not retain the CeO_2_ stoichiometry. Therefore, we employed defect concentrations rather than quantities to calculate their ratios, i.e., [V_O_^••^] = (*n*_V_O_^••^_/(*n*_V_O_^••^_ + *n*_O^2–^_) and [Ce^3+^] = (*n*_Ce^3+^_/(*n*_Ce^3+^_ + *n*_Ce^4+^_).

### 4.3 Identification of Vacancy Oxygen Sites in Optimized Ceria Nanoparticles

The positions of oxygen vacancies V_O_^••^ may change substantially or migrate through neighboring oxygen anions during the structural optimization of nanoparticles. The KLMC-GULP workflow enables the extraction of both the initial and final atomic positions of O^2–^, Ce^4+^, and Ce^3+^, while only the initial positions of V_O_^••^ are available. To obtain physically meaningful coordinates for oxygen vacancies after the structural relaxation of nanoparticle models with randomly introduced defects, we developed an algorithm to locate and refine the positions of V_O_^••^ based on the local coordination environments of CeO_2_.

First, the algorithm assesses the original V_O_^••^ positions by calculating their coordination numbers with O^2–^ in the optimized nanoparticle. If the minimum O^2–^–V_O_^••^ distance falls below 1.35 Å (half of the normal O^2–^-O^2–^ bond length in CeO_2_), the vacancy site is classified as “migrated”, indicating that a neighboring oxygen has filled the site, leading to vacancy relocation in the structural optimization.

Second, the algorithm identifies the new location of the migrated V_O_^••^ by screening the set of original positions of the original O^2–^ ions within a 3.55 Å radius of the “migrated” vacancy sites (including only nearest neighboring sites). Each candidate site is assessed based on its shortest distance to surrounding oxygen ions in the optimized structure. The site characterized by the longest O^2–^–V_O_^••^ minimal distance was identified as the new vacancy location following migration. This criterion ensures that the newly assigned vacancy site is positioned in the most oxygen-deficient region within the local environment. This step yields a refined list of V_O_^••^ that includes both migrated and stationary vacancies within the nanoparticle’s original coordinate framework.

Third, the algorithm optimizes the V_O_^••^ positions in the context of the relaxed atomic environment. For each vacancy, the first-shell coordination number with Ce ions is determined within a 3.3 Å radius. The refinement strategy then follows two cases: (i) For V_O_^••^ sites with a coordination number of four with Ce, corresponding to bulk-like environments, the algorithm maximizes the average V_O_^••^–Ce distance to define the center of the oxygen vacancy; (ii) For undercoordinated V_O_^••^ sites, corresponding to surface environments, the algorithm enforces an average V_O_^••^–Ce distance of 2.4 Å (approximate bond length of V_O_^••^–Ce^3+^/Ce^4+^) while maximizing the nearest-neighbor V_O_^••^–O distance. This step further ensures a balanced spatial distribution of the vacancy near the surface with physically plausible bond lengths and coordination. The final positions are obtained through constrained numerical optimization using the limited-memory BFGS method, with a maximum displacement of 1.0 Å imposed to avoid unphysical shifts. This algorithm ensures the accurate identification of the V_O_^••^ positions in relaxed ceria nanostructures, thereby mitigating artifacts arising from vacancy migration during structural relaxation.

### 4.4 Synthesis of Ceria Nanoparticles

CeO_2_ nanoparticles were obtained by the continuous hydrothermal method as described elsewhere.^41^ Briefly, the Ce­(IV) octanoate complex was used as the precursor of the reaction, and octanoic acid modified CeO_2_ nanoparticles were obtained under 340 °C, 30 MPa, at the residence time of 0.04, 7.6, or 95 s to obtain different sizes of particles, e.g., 1.9, 3.3, and 6.4 nm (evaluated by TEM). As published previously,^41^ the ultrasmall CeO_2_ was well crystallized as the fluorite structure (*Fm* 3̅ *m, S.G. 225*) and homogeneous in size and shape.

Octanoic acid exhibits a strong affinity for the (100) facets of CeO_2_, thereby stabilizing a predominantly cubic morphology in the synthesized nanoparticles.^41^ However, as the particle size decreases below approximately 5 nm, additional facets such as (111) and (110) become increasingly prominent, leading to a truncated cubic shape. Following the removal of the octanoic acid modifier through calcination at 300 °C for 2 h, compositional measurements were performed. The cerium content was quantified using inductively coupled plasma (ICP) analysis, while oxygen content was determined via high-temperature melting and infrared (IR) spectroscopy methods employing a helium carrier (Table S1). The results confirm that the surfaces are predominantly terminated with oxygen atoms. Contrary to expectations of substantial oxygen vacancy formation, the data indicate that oxygen defects are relatively limited after the calcination treatment.

### 4.5 STEM Imaging

Fine powders of ceria were dispersed in ethanol, and a few drops of the suspension were placed onto ultrathin carbon-coated copper grids for STEM imaging. STEM images were collected at 300 kV on a JEM-ARM300F microscope (JEOL) equipped with a cold field emission gun (FEG) and double Cs correctors. The convergence semi-angle is 24 mrad and the collection angle is 27–110 mrad for ADF images, 5.9–12 mrad for ABF images.

### 4.6 Synchrotron XPS Spectroscopy

The synchrotron XPS was conducted at beamline BL07LSU at SPring-8.^80,81^ The organic modified CeO_2_ samples were pelletized without any treatment and loaded on the holder in the chamber. Two different incident X-rays, e.g., 1070 and 1800 eV, were used by the Monk–Gillieson mounting monochromator.^80^ Ce 3d spectra were obtained for two different CeO_2_ nanoparticles. The incident angle of the X-ray and the emission angle of photoelectrons were 34.8° and 77.2° with respect to the surface, respectively.

## Supplementary Material



## Notes

Supporting data on hybrid QM/MM results on formation energies of intrinsic defects, self-consistent Fermi levels, thermodynamic defect concentrations, predicted oxygen nonstoichiometry in CeO_2_, size and composition of synthesized ceria nanoparticles, probing depths in synchrotron X-ray techniques, fitting results for the XPS data, and Monte Carlo simulation results (PDF)
