# Supplementary material for: Surface-Driven Electron Localization and Defect Heterogeneity in Ceria
Source: J Am Chem Soc. 2025 Sep 9;147(37):33888–902. doi: 10.1021/jacs.5c10679 (PMC12447479; doi:10.1021/jacs.5c10679)
Supplement: Supplementary file 1 [file ja5c10679_si_001.pdf]

Supporting Information for

Surface-Driven Electron Localisation and Defect Heterogeneity in Ceria

Xingfan Zhang[a, *], Akira Yoko[b, c], Yi Zhou[d], Woongkyu Jee[a], Alvaro Mayoral[g,d], Taifeng Liu[a, h], Jingcheng Guan[a], You Lu[e], Thomas W. Keal[e], John Buckeridge[f], Kakeru Ninomiya[c], Maiko Nishibori[c,j], Susumu Yamamoto[c,j], Iwao Matsuda[c,k], Tadafumi Adschiri[b,i], Osamu Terasaki[d,i], Scott M. Woodley[a], C. Richard A. Catlow[a, l, *], and Alexey A. Sokol[a, *]

[a] Kathleen Lonsdale Materials Chemistry, Department of Chemistry, University College London, London WC1H 0AJ, United Kingdom.

[b] WPI-Advanced Institute for Materials Research (WPI-AIMR), Tohoku University, 2-1-1 Katahira, Aoba-ku, Sendai 980-8577, Japan.

[c] International Center for Synchrotron Radiation Innovation Smart (SRIS), Tohoku University, 468-1, Aramaki-Aza-Aoba, Aoba-ku, Sendai 980-8572, Japan.

[d] Centre for High-Resolution Electron Microscopy (CħEM), School of Physical Science and Technology and Shanghai Key Laboratory of High-Resolution Electron Microscopy, ShanghaiTech University, Shanghai 201210, P. R. China.

[e] Scientific Computing Department, STFC Daresbury Laboratory, Warrington, Cheshire WA4 4AD, United Kingdom.

[f] School of Engineering and Design, London South Bank University, London SE1 OAA, United Kingdom.

[g] Instituto de Nanociencia y Materiales de Aragón (INMA), CSIC-Universidad de Zaragoza, Zaragoza 50009, Spain.

[h] National & Local Joint Engineering Research Center for Applied Technology of Hybrid Nanomaterials, Henan University, Kaifeng 475004, China.

[i] New Industry Creation Hatchery Center, Tohoku University, Sendai 980-8579, Japan.

[j] Institute of Multidisciplinary Research for Advanced Materials, Tohoku University, Sendai 980-8577, Japan.

[k] The Institute for Solid State Physics, The University of Tokyo, Kashiwa, Chiba 277-8581, Japan.

[l] School of Chemistry, Cardiff University, Park Place, Cardiff CF10 1AT, United Kingdom.

Corresponding authors:

* xingfan.zhang.20@ucl.ac.uk; c.r.a.catlow@ucl.ac.uk; a.sokol@ucl.ac.uk

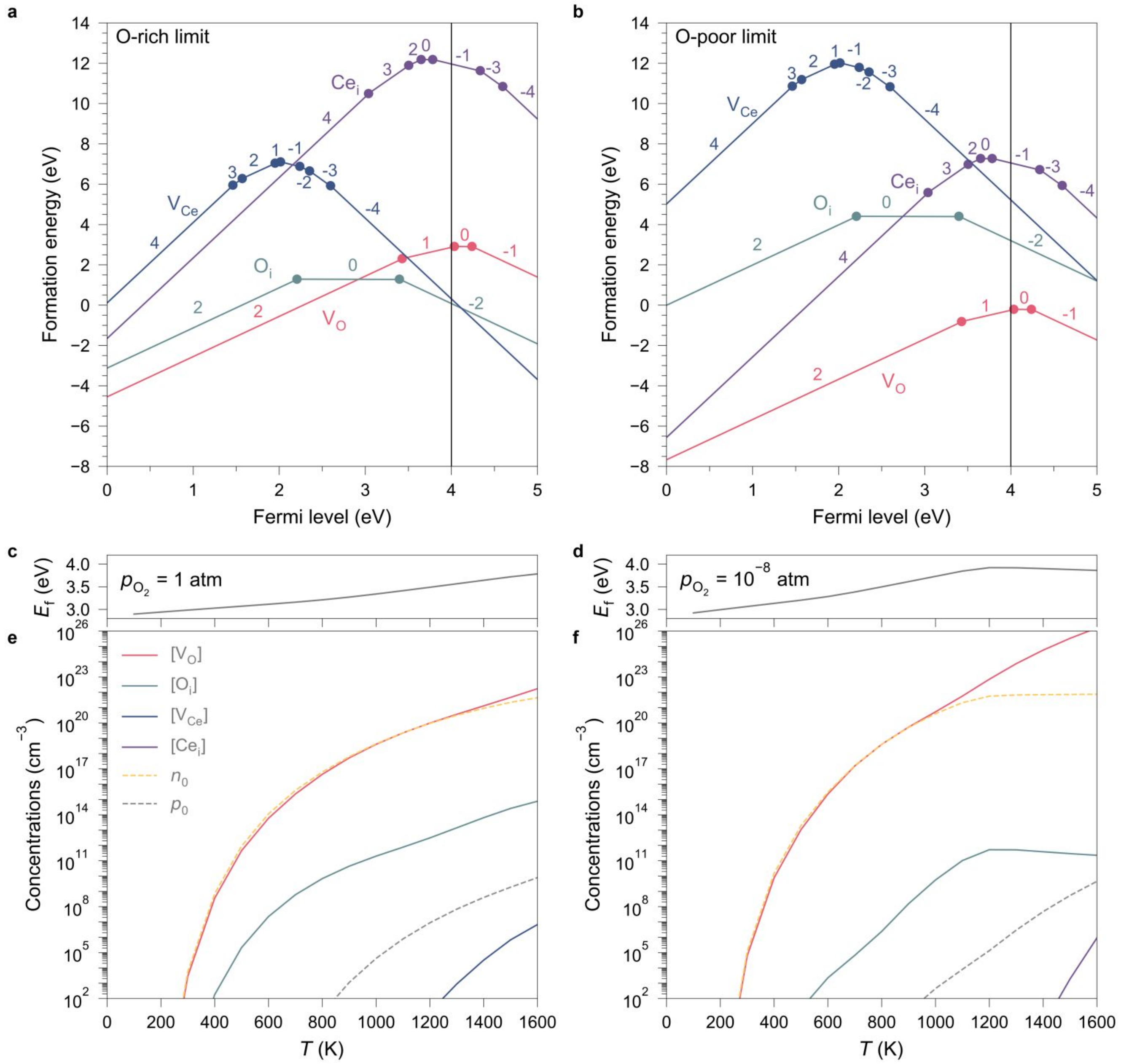

Figure S1. Intrinsic defect chemistry in bulk CeO_2 calculated by the hybrid QM/MM approach with the BB1K hybrid functional. Formation energies of point defects in different charge states as a function of the Fermi level in (a) O-rich and (b) O-poor limits. Thermodynamic transition levels were shown as solid circles. The predicted self-consistent Fermi levels and concentrations of defects and charge carriers in bulk CeO_2 at varying temperatures with oxygen partial pressures of (c, e) 1 atm and (d, f) 10^{-8} atm.

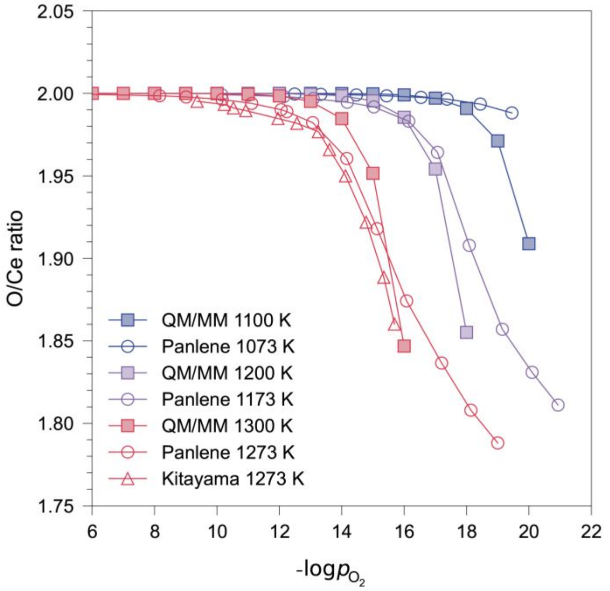

Figure S2. Oxygen nonstoichiometry in bulk ceria under varying reduction conditions predicted by the QM/MM embedded-cluster models and compared with experimental data. Experimental results are adapted from Panlene et al.[1] and Kitayama et al.[2]

a

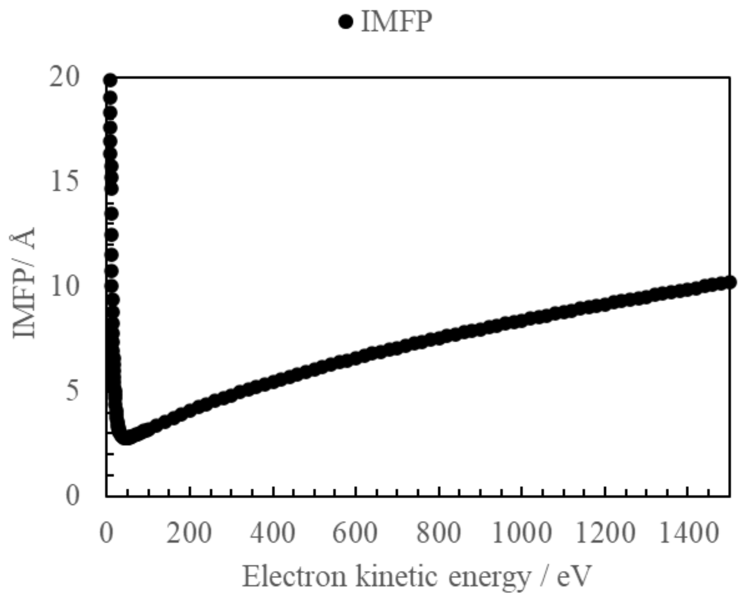

b

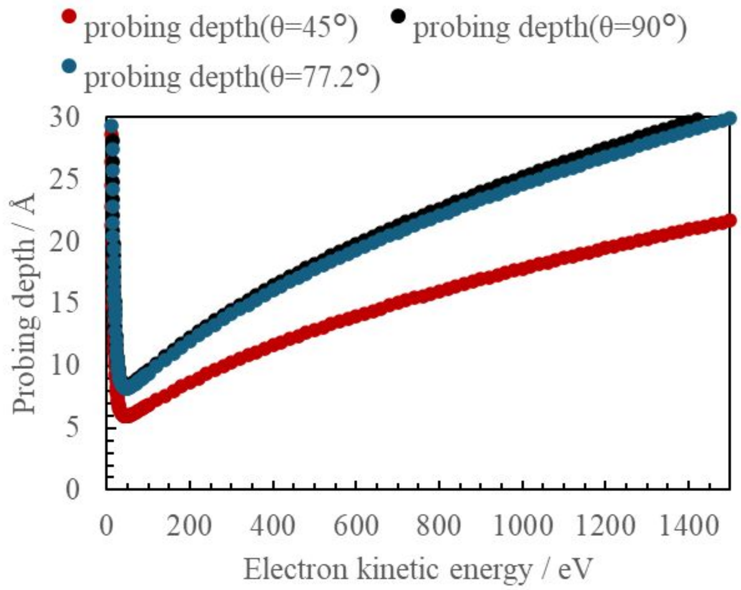

Figure S3. (a) Inelastic mean free path (IMFP) of synchrotron X-ray techniques and (b) simulated probing depth using different electron kinetic energies.

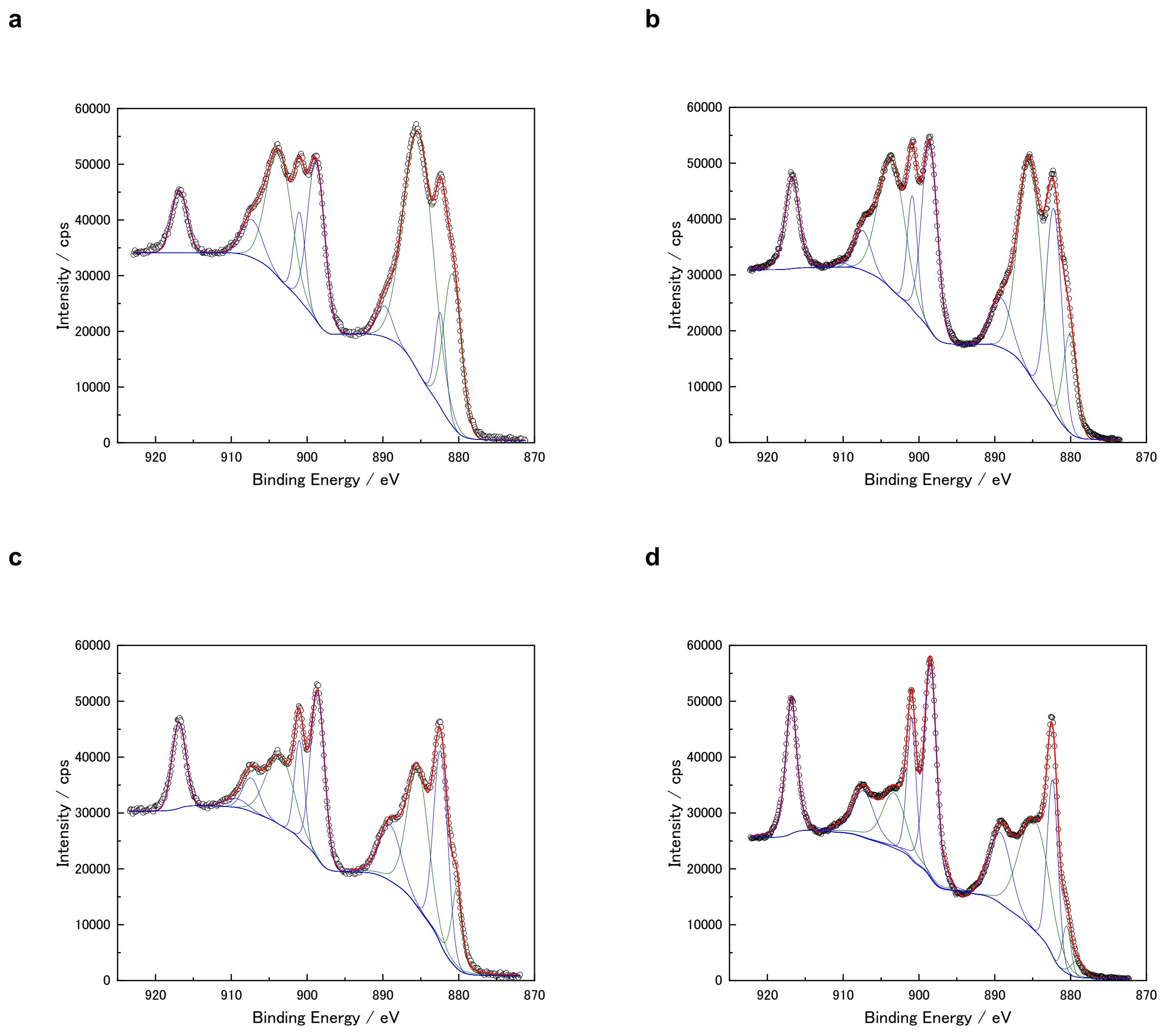

Figure S4. Fitting results for the XPS data (a) 1.9 nm CeO_2 1070 eV incident X-rays, (b) 1.9 nm CeO_2 1800 eV incident X-rays, (c) 6.4 nm CeO_2 1070 eV incident X-rays, (d) 6.4 nm CeO_2 1800 eV incident X-rays. Ce 3d XPS spectra were curve-fitted using a Gauss function with the Shirley background.

Table S1. Measured sizes and compositions of ceria nanoparticles.

Particle size (nm)	Ce wt%	O wt%	O/Ce atomic ratio	Note
3	78.7	21.5	2.39	Synthesized by supercritical method in this study
5	79.0	20.2	2.24	Synthesized by supercritical method in this study
7	79.0	21.3	2.36	Synthesized by supercritical method in this study
8	78.7	20.2	2.24	Synthesized by supercritical method in this study
25	82.5	18.9	2.01	Reference purchased from Sigma Aldrich

Table S2. Percentages of the peak intensities of Ce^{3+}.

	1070 eV (Surface)	1800 eV (Bulk)
1.9 nm	64.9 %	48.3 %
6.4 nm	39.3 %	34.2 %

Table S3. Relative concentrations of $V_O^{\bullet\bullet}$ and Ce^{3+} ions in the bulk and surface regions of ceria nanoparticles, as predicted by unbiased Monte Carlo simulations using intrinsically nonstoichiometric models, where initial models retain the surface structure of CeO_2 and incorporate additional Ce^{3+} to preserve overall charge neutrality. The values are averaged from the lowest-energy 1,000 configurations among 10,000 sampled configurations. κ denotes the local ratio $[Ce^{3+}]/2[V_O^{\bullet\bullet}]$ as defined by eq. (2) in the main text. The data correspond to the results shown in Figure 3c in the main text.

Shape	$[V_O^{\bullet\bullet}]$ (total)	$[Ce^{3+}]$ (bulk)	$[Ce^{3+}]$ (surf)	$[V_O^{\bullet\bullet}]$ (bulk)	$[V_O^{\bullet\bullet}]$ (surf)	κ (bulk)	κ (surf)	κ (total)
Octa	1%	9.34%	10.39%	0.99%	0.97%	4.866	6.931	5.123
Octa	5%	25.12%	26.25%	4.95%	5.01%	2.548	2.716	2.569
Octa	10%	45.05%	45.79%	10.00%	9.95%	2.257	2.340	2.268
Octa	15%	64.62%	65.32%	14.90%	15.09%	2.171	2.190	2.169
Trun	1%	9.10%	10.64%	1.02%	0.90%	4.571	7.600	5.001
Trun	5%	25.80%	25.54%	4.99%	5.02%	2.598	2.661	2.567
Trun	10%	45.70%	45.03%	10.00%	9.99%	2.290	2.299	2.269
Trun	15%	65.43%	64.70%	14.97%	15.08%	2.189	2.172	2.169
Cube	1%	9.79%	10.68%	0.97%	1.05%	5.100	6.306	5.119
Cube	5%	25.46%	26.74%	4.96%	5.16%	2.574	2.684	2.593
Cube	10%	45.16%	46.35%	9.97%	10.10%	2.267	2.332	2.281
Cube	15%	64.98%	65.79%	14.97%	15.10%	2.171	2.202	2.177

Table S4. Relative concentrations of $V_O^{\bullet\bullet}$ and Ce^{3+} ions in the bulk and surface regions of ceria nanoparticles, as predicted by unbiased Monte Carlo simulations using intrinsically nonstoichiometric, octahedral models with different sizes, with a fixed $[V_O^{\bullet\bullet}]$ of 5%. The data correspond to the results shown in Figure 3d.

r (Å)	$[V_O^{\bullet\bullet}]$ (total)	$[Ce^{3+}]$ (bulk)	$[Ce^{3+}]$ (surf)	$[V_O^{\bullet\bullet}]$ (bulk)	$[V_O^{\bullet\bullet}]$ (surf)	κ (bulk)	κ (surf)	κ (total)
5	5%	41.06%	42.72%	5.18%	4.82%	4.619	5.312	4.235
7	5%	30.78%	31.39%	4.94%	4.86%	3.196	3.528	3.174
9	5%	27.04%	26.95%	5.04%	4.91%	2.704	2.892	2.699
11	5%	25.80%	25.54%	4.99%	5.02%	2.598	2.661	2.567

References

(1) Panlener, R.; Blumenthal, R.; Garnier, J. A thermodynamic study of nonstoichiometric cerium dioxide. *J. Phys. Chem. Solids* **1975**, *36* (11), 1213-1222.

(2) Kitayama, K.; Nojiri, K.; Sugihara, T.; Katsura, T. Phase equilibria in the CeO and CeFeO systems. *J. Solid State Chem.* **1985**, *56* (1), 1-11.
